# Feasibility of an Interactive Patient Portal for Monitoring Physical Activity, Remote Symptom Reporting, and Patient Education in Oncology: Qualitative Study

**DOI:** 10.2196/15539

**Published:** 2019-11-28

**Authors:** Michael Marthick, Anna Janssen, Birinder S Cheema, Jennifer Alison, Tim Shaw, Haryana Dhillon

**Affiliations:** 1 Research in Implementation Science and eHealth Group Faculty of Medicine and Health University of Sydney Camperdown Australia; 2 Department of Supportive Care and Integrative Oncology Chris O'Brien Lifehouse Camperdown Australia; 3 School of Science and Health University of Western Sydney Penrith Australia; 4 Sydney Local Health District Sydney Australia; 5 Department of Health Sciences University of Sydney Lidcombe Australia; 6 Centre for Medical Psychology & Evidence-based Decision-making School of Psychology University of Sydney Camperdown Australia

**Keywords:** physical activity, patient Web portals, neoplasms

## Abstract

**Background:**

Digital health interventions, such as the use of patient portals, have been shown to offer benefits to a range of patients including those with a diagnosis of cancer.

**Objective:**

This study aimed to explore the participant experience and perception of using an interactive Web-based portal for monitoring physical activity, remote symptom reporting, and delivering educational components.

**Methods:**

Participants who were currently under treatment or had recently completed intensive treatment for cancer were recruited to three cohorts and invited to join a Web-based portal to enhance their physical activity. Cohort 1 received Web portal access and an activity monitor; cohort 2 had additional summative messaging; and cohort 3 had additional personalized health coaching messaging. Following the 10-week intervention, participants were invited to participate in a semistructured interview. Interview recordings were transcribed and evaluated using qualitative thematic analysis.

**Results:**

A total of 17 semistructured interviews were carried out. Participants indicated that using the Web portal was feasible. Personalized messaging improved participant perceptions of the value of the intervention. There was a contrast between cohorts and levels of engagement with increasing health professional contact leading to an increase in engagement. Educational material needs to be tailored to the participants’ cancer treatment status, health literacy, and background.

**Conclusions:**

Participants reported an overall positive experience using the Web portal and that personalized messaging positively impacted on their health behaviors. Future studies should focus more on design of interventions, ensuring appropriate tailoring of information and personalization of behavioral support messaging.

**International Registered Report Identifier (IRRID):**

RR2-10.2196/9586

## Introduction

### Background

Digital health interventions may more effectively engage cancer patients to self-manage health-related concerns and behavior change [1]. The feasibility and effectiveness of Web portals have been tested in a variety of cohorts with chronic disease and may provide an opportunity to improve the delivery of cancer care [2]. Web portals have been demonstrated to offer a range of benefits to patients. It has been shown that patients with chronic diseases with access to Web portals have greater engagement in their treatment, lower levels of treatment-related distress, increased treatment satisfaction, and improved communication with health professionals [2-6]. However, the use of Web portals to support a multicomponent program of physical activity behavior change, remote symptom monitoring, and delivery of supportive care education for people with cancer has not been evaluated.

Physical activity levels vary throughout treatment and beyond in people diagnosed with cancer. Typically, physical activity decreases throughout and following intensive treatment such as chemotherapy, and commonly fails to reach prediagnosis levels [7,8]. This reduction in physical activity levels negatively impacts upon health status, including numerous treatment-related side effects and potentially mortality [9-11].

To support patients in an Australian comprehensive cancer center, we developed and piloted an interactive Web portal to support physical activity behavior change and symptom monitoring [12]. A range of features was available through the Web portal dependent on the cohort to which the patient was allocated. We have previously reported that feasibility and acceptability criteria were met, with engagement increasing with more feedback and health professional contact and was highest in those participants who received individual personalized messaging [12]. To provide greater depth of understanding of the patients’ experiences and perceptions of the Web portal, semistructured interviews were needed.

### Objectives

In this analysis, we aimed to explore participants’ experiences with the Web portal and their perceptions of its impact on their physical activity behavior. It was achieved through the use of semistructured interviews with participants following the 10-week intervention.

## Methods

### Study Design

**Figure 1:**
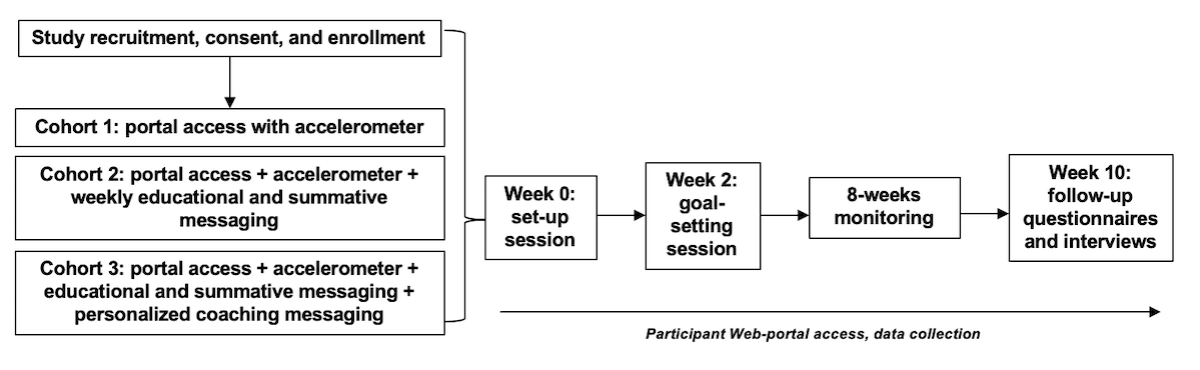
Study procedures.

This nested qualitative substudy was part of a larger feasibility study of a digital health care intervention for people with a history of cancer [12]. The intervention was developed utilizing evidence-based components of education, goal setting, monitoring, feedback, and motivation underpinned by the theoretical framework from Michie et al [13] and the transtheoretical model of behavior change [14]. Personalized health coaching elements were designed to deliver a motivational interviewing style intervention through a remote delivery platform [15]. Participants who had recently completed intensive anticancer therapy, and who were over 18 years of age and English speaking were recruited to 1 of 3 cohorts. Cohort 1 was provided access to the Web portal and given a commercially available wearable physical activity and sleep tracker (Misfit Shine) for the intervention period, along with emailed weekly cancer-focused educational material. Cohort 2 was given the same content, with the addition of an emailed weekly message providing participants with a summary of their exercise history, sleep duration, and an overview of their reported symptom scores. Cohort 3 received the same content as cohort 2 plus regular personalized coaching email messages from an accredited exercise physiologist. These messages focused on a range of behavioral change strategies, including motivational, discussed fatigue and pain scores, and provided feedback on and goal targets for step counts. Study procedures are shown in Figure 1.

Following the 10-week intervention, the evaluation of participant use of and engagement with the Web portal was supplemented by in-depth qualitative interviews. A thematic analysis approach was taken.

### Ethics, Consent, and Permissions

All procedures performed in this study were in accordance with the ethical standards of the institutional and national research committee, and with the 1964 Helsinki declaration and its later amendments or comparable ethical standards. Permission to conduct this study was granted by the Royal Prince Alfred Hospital Human Research and Ethics Committee (X16-0051). All participants provided written informed consent.

#### Consent to Publish

All individual participants included in the study provided consent to publish.

### Participant Recruitment

Participants of the substudy were recruited from our previously reported larger cohort study [12]. All participants were either currently undergoing treatment for their cancer, or had completed treatment within the last 6 months, and were recruited from a single cancer care center located in Sydney, Australia. Participants were purposively sampled from across each of the 3 study cohorts, and each of the participants had completed the intervention before participating. Each potential participant was approached by telephone by one of the research team members seeking consent for a semistructured interview. For those consenting, time for a telephone interview was mutually agreed and booked.

### Procedure

Interviews were completed from July to September 2017. Individual semistructured interviews were conducted once only, via telephone from a private meeting room by a PhD-qualified female, translational health researcher with experience in qualitative methods (AJ). The interviewer was not known to participants and not involved in the larger feasibility study. Participants were informed that the discussion was being audio-recorded and verbal confirmation of their consent for this was obtained. Only the participant and researcher were present during interviews. Field notes were recorded during interviews.

Participants were asked to explore their experiences of using the Web portal, using an activity monitor, and their perception of the personalized coaching messages if they were in cohort 3. An overview of the semistructured interview guide is provided in Textbox 1. Questions were intended to guide the conversation, rather than be prescriptive. The interviewer was responsive to participant comments and tailored questions and probes to draw out the informative comments from participants.

Audio files were transcribed verbatim by a professional transcribing service. Accuracy of transcriptions was checked by 1 of 2 authors (MM and AJ) before each being analyzed. Participants were not sent transcripts for comment and did not give feedback on the research findings. Recruitment of participants continued until data saturation was achieved [16]. To limit the possibility of introducing bias, each author had independently reviewed the transcripts and agreed data saturation had been achieved. Those interviews already booked were completed to confirm no new themes were identified. The COnsolidated criteria for REporting Qualitative research checklist is given in Multimedia Appendix 1.

**Interview framework.**

*Initial exploration*

- Could you please tell me about your previous use of technology (eg, applications and fitness trackers)?
- Could you tell me about how you found using the Web portal?
- Usability of the Web portal
- Use and usefulness of the accelerometer
- Quality and usefulness of seeing and inputting your data
- Did you utilize the educational part of the Web portal (eg, nutrition information)?

*For people getting personalized messaging*

- How did you find the weekly personalized messaging/coaching you received?
- Do you recall any specific messages that you received?
- Did the messaging help to motivate you?

*For people getting summative messaging*

- How did you find the weekly summative messaging you received?
- Was the data useful?
- Did the messaging help to motivate you?

*Advice*

- Are there things that could have been done differently that may have improved your experience?
- What would you recommend to other people using the Web portal?
- Will you continue to use the portal?
- Yes. Why?
- No. Why not?

*Future use*

- Explore the concept of gamification—individual and between participants.
- Positives and negatives
- Discuss the use of video calls with health professionals as part of the portal.
- Positives and negatives
- Would you pay to use the Web portal?
- Potential business model

### Data Analysis

We analyzed the interview data thematically [17] using a framework approach [18]. Initially, the interviews were coded line by line for descriptive experiences (MM, male; MPH, health researcher, and exercise physiology clinician). In all, 3 transcripts were distributed for individual review and independent initial code generation among the team (MM; HD, female, PhD, behavioral scientist; and AJ). MM and HD were not present for the participant interviews. After consultation and cross-coding, the initial codes were expanded facilitating development of a coding tree. Data were charted using Microsoft Excel version 16.5 [19]. Attention was paid to contrasting differences in experience between the 3 study cohorts. We then compared and contrasted emergent themes until we were confident that we had captured the predominant thoughts and perspectives evident in the interviews. As a research team, we refined these themes for clarity and completeness. To maintain data rigor and independent coding, consensus, when disagreements arose, was achieved through group discussion, data saturation was determined independently and agreed by researchers, and independent and comparative coding was undertaken.

## Results

A total of 49 participants (median age 54 years, range 28-79, 22% [11/49] male) took part in the feasibility study across the 3 cohorts. Of these, 17 completed semistructured interviews via telephone (median age 57 years, range 30-79, 35% [6/17] male). The median length of interview was 19 min (range 13-26 min). Demographic details of each participant are presented in Table 1.

Themes were iteratively developed from exploratory categories. We purposefully explored 3 areas related to the Web portal. These areas were (1) engagement, (2) design and usability, and (3) future developments. Several subthemes underpinned each of these 3 overarching themes and are detailed in Textbox 2.

**Table 1:** Individual interviewee demographics.

Cohort: participant number^a,b,c^	Age (years)	Sex	Cancer type classification	Stage of treatment (active therapy or survivorship)
1.1	55	F	Breast	Survivorship
1.2	42	F	Breast	Survivorship
1.3	54	M	Melanoma	Active
1.4	75	F	Colorectal	Survivorship
1.5	79	M	Prostate	Survivorship
1.6	59	F	Hematological	Active
1.7	58	F	Breast	Survivorship
2.1	30	M	Hematological	Active
2.2	52	F	Hematological	Active
2.3	60	F	Breast	Survivorship
2.4	61	F	Breast	Active
2.5	57	M	Head and neck	Survivorship
3.1	40	M	Colorectal	Active
3.2	73	F	Breast	Survivorship
3.3	59	F	Breast	Survivorship
3.4	51	M	Hematological	Survivorship
3.5	54	F	Lung	Survivorship

^a^Cohort 1: portal/device only.

^b^Cohort 2: additional automated education.

^c^Cohort 3: additional tailored coaching messaging.

**Overview of qualitative themes.**

- Engagement through intervention
  - Facilitated behavior change
  - Device wearability and engagement
  - Engagement with intervention
  - Impact of personalized messaging
  - Personal factors impacting on engagement
  - Technical issues impacting engagement
- Web portal
  - Patterns of use and engagement
  - Ease of use
  - Educational content
- Future development
  - Gamification
  - Addition of telehealth consultations
  - Developing a business model

### Engagement Through Intervention

The engagement theme encompassed aspects of the Web portal, the wearable device, personalized messaging, and a range of other factors impacting behavior. Many participants, particularly those in cohorts 2 and 3, indicated that the intervention facilitated positive behavior change:

I really liked it. I found that just... It gave me a bit more motivation to actually increase my activity level. I did find that I was checking my wristband a lot to see if I'd met my daily activity goal and when I didn't I felt like... Not a sense of... Not failure but just like, “Aw, I didn't meet my goal. I have to make sure I do extra tomorrow,” kind of thing. It was just really, really motivating. I felt like I did definitely increase my exercise over that time.

Participant 3.1

So that was sort of an on-going, that's what kept me honest.

Participant 3.5

Some days you might be more bound to your desk or at home not doing so much, and it's a good tool to prompt you to change your habits, and get up...

Participant 1.6

The activity tracker (Misfit Shine) provided to most participants at commencement of the intervention was generally well liked. Participant feedback suggests the usability and engagement of this device to be high across each of the 3 cohorts:

Yes. I absolutely love the thing that you wear on your arm. I'm just elated. I think it's really motivating, and I really enjoyed having that.

Participant 1.3

Absolutely, absolutely I loved it. It was really good to see exactly what it took to get to my goal each day and I love it. To the point I'm going to get another one and it's going to be a part of my life to have a fitness tracker now.

Participant 3.3

Overall, participants in all cohorts were generally positive about their involvement, indicating a positive engagement with the intervention as a whole:

I just thought it was a very constructive and positive experience... it was really helpful in terms of making a progressive recovery.

Participant 2.4

It was really good to be part of it, it really helped me through my chemo so I was really grateful.

Participant 3.1

Those participants in cohort 3 who received personalized messaging typically revealed that the use of personalized coaching messaging was highly acceptable and provided additional motivation to help them succeed with goal attainment and increasing physical activity levels:

…and it actually made me happy. It gave me a sense of achievement, especially when the EP would send the message saying, “Wow, you've matched your goals. Well done.” I felt a lot of pride in myself.

Participant 3.1

It made me just push myself and even on days when I didn't want to walk I thought no my steps were down and I should get out there and go for a walk and so on.

Participant 3.3

They contrasted with cohorts 1 and 2, where participants indicated a need and preference for increase in health professional contact during the intervention period:

…but if someone motivated me to say, “Would you like to come in and have a look at that app again and I'll show you what it does. And let's see how you're going with it,” then that might have...I might have engaged with it a bit more...or at all.

Participant 1.3

...some interaction and discussion with the individual (researcher), I would think that that would improve uptake, it would also encourage you to think about it a bit more.

Participant 2.6

And I guess that I don't interact with anybody or get anything but a lot of information that I already know doesn't add much to that undertaking. If it was a more interactive component maybe or something.

Participant 1.1

Personal factors appeared to have an impact on engagement. The intervention gave 1 participant an opportunity to reassure their family about the safety of their exercise plan:

They worried about me overdoing it, that sort of thing. And so I could go back to them and say, “Look, I've spoken to the exercise physiologists at the hospital and they say this is okay...” I think for them, they were reassured, as far as they're concerned.

Participant 3.2

A small subset of participants had technical issues with the perceived accuracy of their device; in particular, they felt the sleep tracking was inaccurate. It appeared to impact their use of the Web portal and decreased their engagement in remote monitoring:

I think the sleep is inaccurate, I think definitely the exercise and the physical activity was probably much more accurate.

Participant 3.5

So I don't think the data is accurate, so I didn't bother.

Participant 2.3

### Web Portal

Focusing on the Web portals’ design and usability, we identified 3 additional themes, as described in Textbox 2. They included patterns of use and engagement. Reported use varied among participants, from a daily habit to less frequent interactions. Overall, participants in cohorts 2 and 3 reported engaging more with the Web portal than those in cohort 1:

I used it daily to do the updates. It's really easy to use, it's to the point and I thought it was really good the way it gathers information to the quantities of data.

Participant 2.4

Weekly, just weekly. I went in, I put my data in daily and did my weekly update, and then I went into and also read the articles weekly.

Participant 2.2

Participants in cohort 1, who had no messaging or health professional interactions, reported engaging with logging their symptoms and viewing content early in the program. However, this was more likely to decrease over time when compared with cohorts 2 and 3:

…and I did go onto the portal a few times, but I haven't been on it, I'd say, the last couple of weeks.

Participant 1.6

Reported ease of use of the Web portal was also seen to be impacted with less health professional interaction. A number of participants in cohort 1 who had no additional interaction following the goal attainment session reported more technological barriers:

I could have done with a few more lessons in how it worked, because I know how to collect the steps and how to log on. But maybe another session in just following up on showing me ...So I feel like if someone had said, “Would you like [me to]...Check on how you're going with it and show you some other things that are available,” that probably would help.

Participant 1.3

Cohorts 2 and 3 had access to a curated selection of Web portal educational content which was also sent in weekly emails, focused on supportive cancer care–specific topics such as sleep, fatigue, and nutrition. It included written articles, video content, and links to government-supported information. Participants typically found the Web portal educational content to be acceptable:

I like to be able to look at and research more information and have different resources available. So I did find that quite useful.

Participant 2.6

I thought it was really good, the information was presented in a glaring manner.

Participant 2.4

In the main study, the percentage of participants who opened a link in their educational email averaged 60% to 70% each week. It ranged from 59% to 94% depending on the week and topic area [12]. When probed in interviews, we identified a need to tailor content to the stage of participants’ cancer treatment:

Some of the stuff I might have been interested in two and a half years ago, but it's not so relevant to me now.

Participant 3.5

If I was sort of in the middle of cancer treatments, like active cancer treatment, I probably would have found the information more helpful.

Participant 2.2

Respondents found the educational content too broad and basic; they expressed a preference for more specific, detailed information:

I looked at it once or twice, but I just found it a bit basic.

Participant 2.5

A lot what was written was things that I had read already. That's why I wasn't finding a whole lot of new information for me.

Participant 2. 2

### Future Developments

The third major theme was related to future developments of the Web portal, with 3 subthemes identified. Results indicated variability in preferences and that individual tailoring is required across the care continuum. There were some positive responses to gamification:

I was always really, really interested to know how I was doing compared to the other patients. Not necessarily specifically but just like, “You're in the top 5% of the patients,” or something like that. That would have been really, really... Even more motivating to know how well I was doing in general, compared to the other people for sure.

Participant 3.2

While others had concerns about the impact of gamification on individual sense of achievement and ongoing motivation:

Possibly not because I've problems with walking long distances, and it would perhaps make me feel more self-conscious that I couldn't actually achieve what other people achieve.

Participant 1.6

Several participants welcomed the possibility of commercialization of such an intervention, although engagement is likely to be dependent on pricing:

I don't think anyone really likes to pay, but depending on how much it was, I would.

Participant 3.4

I'm not sure because I am, as I said, because I'm at a different stage of my experience with cancer that, had I'd been in the middle of it, I would probably feel differently. At this stage, probably not.

Participant 2.5

We also discussed the use of video calls as a supplement to the program. Again, there was a mixed response to this concept, and engagement would likely vary across the population:

Absolutely actually...Particularly questions on fitness and questions on nutrition, yeah. Because they're the hard ones to get, right? There's not enough of them at the hospital, to be honest.

Participant 2.3

Probably not. Sounds a bit too much like work.

Participant 1.6

## Discussion

### Principal Findings

The main findings of this study are that (1) participants reported increased health professional contact facilitated greater engagement in the Web portal, (2) participants perceived benefit in using the provided activity tracker (Misfit Shine), and (3) that education, support, and feedback mechanisms need to be specifically tailored to each individual. These findings support our earlier findings around the feasibility and acceptability of a Web portal and activity tracking in a mixed population of cancer survivors [12]. Here our participants’ experiences have provided novel, in-depth perspectives on the usability of a clinician-patient Web portal.

One of our key themes indicates that oncology health support programs and systems should be tailored using a market fragmentation approach, a concept that there is diversity within all markets and each market is composed of multiple segments (eg, individual patients), reflecting different needs, behaviors, and responses to engagement within users [20]. This approach in cancer care enables health support programs and systems to be tailored to different needs and preferences of individual patients and survivors. Previous digital health interventions have typically not done this. Therefore, high rates of dropouts and low engagement are the results.

Supporting our quantitative data, qualitative results indicated a contrast among the 3 cohorts and their levels of engagement with the Web portal. It supports the conclusion that increasing health professional contact led to an increase in reported engagement. The study highlights the importance of personalized messaging and tailoring information to increase participant perceptions of the value of the intervention.

This study highlighted the key theme of matching an individual with an appropriate feedback strategy when implementing these types of interventions, which includes the use of personalized messaging. There has been a recent interest, and promising results, in the use of automated messaging, such as those sent through SMS, to drive health behavior change within certain populations, such as for people with diabetes and depression [21,22]. Although there is potential for automated interventions be delivered to 1 patient subgroup with positive effect, others may best respond to personalized messaging sent by a health professional or health coach. This approach to targeting population via tailored messaging requires more research to be used effectively in practice.

This study also emphasized the critical need for tailored educational material congruent with individual’s health literacy level, prior health knowledge, treatment status, and prognosis. This finding is supported by a recent systematic review across multiple chronic conditions [23], which concluded that there was a moderate level of evidence supporting tailoring of communication strategies to patient health literacy. Several other studies have reported positive results when tailoring communications to different stages of the cancer care trajectory, as there are evolving information needs across the cancer care trajectory with those needs being quite distinct in active treatment compared with survivorship phases of care [24-26]. It is noted that our study included both participants undergoing intensive cancer treatment and those in the survivorship stage. They need to be differentiated.

Although the intervention participants took part in did not include any concepts of gamification, interview questions explored whether this would have any additional benefit. Gamification focuses on applying game mechanics to nongame contexts to improve engagement and support lasting change [27]. There were mixed responses to the role of gamification in this study ranging from enthusiastic support through to concerns that it would negatively impact those who were unable to *compete* fully because of physical side effects of treatment. Previous *corporate* health and wellness offerings have included such concepts as challenges, points, leader boards, and rewards mechanisms [28-30]. These concepts are emerging in the health care field [31,32]; however, such interventions may have challenges adapting to the specific needs of patients during intensive cancer therapy, or who have recently completed such treatment. There may also be potential concerns regarding privacy legislation [27]. This concept requires further investigation.

If there are promising results, digital health interventions need to consider scalability, and how they could be widely enabled within a health system. In the case of this intervention, patient access to Web portals, enhanced with components such as personalized coaching messaging, has the potential for broader dissemination.

### Limitations

The study has some limitations. As a nested substudy, we recruited only a subset of those participants from our larger feasibility study, and their experience may not be representative of all study participants or the broader cancer population. We used data saturation to determine when to cease recruitment to the study, and this may have introduced a bias through the interpretation of interview data by the research team. Given the predominance of women with breast cancer in our study, the results may be biased by their reported perceptions over other tumor groups. Participants were largely white living in metropolitan areas, which does not provide insight into the needs of culturally and linguistically diverse or regional and rural populations.

### Conclusions

With an increasing interest in, and use of, digital interventions in supportive cancer care, there is a need to understand patient experience of such technology. Our participants reported a mostly positive experience of using a Web portal and activity monitor. It was also clear that personalized messaging positively impacted on participants’ health behaviors. Future studies should focus more on design of interventions, ensuring appropriate tailoring of information and personalization of behavioral support messaging.

## Appendix

Multimedia Appendix 1 COnsolidated criteria for REporting Qualitative studies (COREQ): 32-item checklist.

## References

[1] Escriva Boulley G, Leroy T, Bernetière C, Paquienseguy F, Desfriches-Doria O, Préau M (2018). Digital health interventions to help living with cancer: a systematic review of participants' engagement and psychosocial effects. Psychooncology.

[2] Groen WG, Kuijpers W, Oldenburg HS, Wouters MW, Aaronson NK, van Harten WH (2017). Supporting lung cancer patients with an interactive patient portal: feasibility study. JMIR Cancer.

[3] Kruse CS, Argueta DA, Lopez L, Nair A (2015). Patient and provider attitudes toward the use of patient portals for the management of chronic disease: a systematic review. J Med Internet Res.

[4] Kuijpers W, Groen WG, Oldenburg HS, Wouters MW, Aaronson NK, van Harten WH (2016). eHealth for breast cancer survivors: use, feasibility and impact of an interactive portal. JMIR Cancer.

[5] Kuijpers W, Groen WG, Loos R, Oldenburg HS, Wouters MW, Aaronson NK, van Harten WH (2015). An interactive portal to empower cancer survivors: a qualitative study on user expectations. Support Care Cancer.

[6] Irizarry T, Dabbs AD, Curran CR (2015). Patient portals and patient engagement: a state of the science review. J Med Internet Res.

[7] Courneya KS, Friedenreich CM (1997). Relationship between exercise pattern across the cancer experience and current quality of life in colorectal cancer survivors. J Altern Complement Med.

[8] Irwin ML, Crumley D, McTiernan A, Bernstein L, Baumgartner R, Gilliland FD, Kriska A, Ballard-Barbash R (2003). Physical activity levels before and after a diagnosis of breast carcinoma: the Health, Eating, Activity, and Lifestyle (HEAL) study. Cancer.

[9] van Waart H, Stuiver MM, van Harten WH, Geleijn E, Kieffer JM, Buffart LM, de Maaker-Berkhof M, Boven E, Schrama J, Geenen MM, Terwogt JM, van Bochove A, Lustig V, van den Heiligenberg SM, Smorenburg CH, Hellendoorn-van Vreeswijk JA, Sonke GS, Aaronson NK (2015). Effect of low-intensity physical activity and moderate- to high-intensity physical exercise during adjuvant chemotherapy on physical fitness, fatigue, and chemotherapy completion rates: results of the PACES randomized clinical trial. J Clin Oncol.

[10] Witlox L, Hiensch AE, Velthuis MJ, Bisschop CN, Los M, Erdkamp FL, Bloemendal HJ, Verhaar M, Huinink D, van der Wall E, Peeters PH, May AM (2018). Four-year effects of exercise on fatigue and physical activity in patients with cancer. BMC Med.

[11] Cormie P, Zopf EM, Zhang X, Schmitz KH (2017). The impact of exercise on cancer mortality, recurrence, and treatment-related adverse effects. Epidemiol Rev.

[12] Marthick M, Dhillon HM, Alison JA, Cheema BS, Shaw T (2018). An interactive web portal for tracking oncology patient physical activity and symptoms: prospective cohort study. JMIR Cancer.

[13] Michie S, Richardson M, Johnston M, Abraham C, Francis J, Hardeman W, Eccles MP, Cane J, Wood CE (2013). The behavior change technique taxonomy (v1) of 93 hierarchically clustered techniques: building an international consensus for the reporting of behavior change interventions. Ann Behav Med.

[14] Prochaska JO, DiClemente CC (1983). Stages and processes of self-change of smoking: toward an integrative model of change. J Consult Clin Psychol.

[15] Rubak S, Sandbaek A, Lauritzen T, Christensen B (2005). Motivational interviewing: a systematic review and meta-analysis. Br J Gen Pract.

[16] Saunders B, Sim J, Kingstone T, Baker S, Waterfield J, Bartlam B, Burroughs H, Jinks C (2018). Saturation in qualitative research: exploring its conceptualization and operationalization. Qual Quant.

[17] Braun V, Clarke V (2006). Using thematic analysis in psychology. Qual Res Psycol.

[18] Ritchie J (2013). Qualitative Research Practice: A Guide For Social Science Students And Researchers.

[19] Microsoft Office.

[20] (2018). Monash University.

[21] Arambepola C, Ricci-Cabello I, Manikavasagam P, Roberts N, French DP, Farmer A (2016). The impact of automated brief messages promoting lifestyle changes delivered via mobile devices to people with type 2 diabetes: a systematic literature review and meta-analysis of controlled trials. J Med Internet Res.

[22] Aguilera A, Bruehlman-Senecal E, Demasi O, Avila P (2017). Automated text messaging as an adjunct to cognitive behavioral therapy for depression: a clinical trial. J Med Internet Res.

[23] Schapira MM, Swartz S, Ganschow PS, Jacobs EA, Neuner JM, Walker CM, Fletcher KE (2017). Tailoring educational and behavioral interventions to level of health literacy: a systematic review. MDM Policy Pract.

[24] Shim E, Park JE, Yi M, Jung D, Lee K, Hahm B (2016). Tailoring communications to the evolving needs of patients throughout the cancer care trajectory: a qualitative exploration with breast cancer patients. BMC Womens Health.

[25] Brattheim B, Sand K, Gilstad H, Stalsberg R, Lundgren S, Reidunsdatter RJ (2017). Breast Cancer Patients’ Experiences with Information and Communication in Cancer Disease Trajectories. Proceedings of the 4th European Workshop on Practical Aspects of Health Informatics.

[26] Tan AS, Nagler RH, Hornik RC, DeMichele A (2015). Evolving information needs among colon, breast, and prostate cancer survivors: results from a longitudinal mixed-effects analysis. Cancer Epidemiol Biomarkers Prev.

[27] Sardi L, Idri A, Fernández-Alemán JL (2017). A systematic review of gamification in e-Health. J Biomed Inform.

[28] Steigner G, Doarn CR, Schütte M, Matusiewicz D, Thielscher C (2017). Health applications for corporate health management. Telemed J E Health.

[29] Glance D, Ooi E, Berman YE, Glance CF, Barrett HR (2016). Impact of a Digital Activity Tracker-Based Workplace Activity Program on Health and Wellbeing. Proceedings of the 6th International Conference on Digital Health Conference.

[30] Gawley R, Morrow C, Chan H, Lindsay R (2016). BitRun: Gamification of Health Data from Fitbit® Activity Trackers. Proceedings of the International Conference on IoT Technologies for HealthCare.

[31] Looyestyn J, Kernot J, Boshoff K, Ryan J, Edney S, Maher C (2017). Does gamification increase engagement with online programs? A systematic review. PLoS One.

[32] Alahäivälä T, Oinas-Kukkonen H (2016). Understanding persuasion contexts in health gamification: a systematic analysis of gamified health behavior change support systems literature. Int J Med Inform.

